# Urinary acrolein protein conjugates-to-creatinine ratio is positively associated with diabetic peripheral neuropathy in patients with type 2 diabetes mellitus

**DOI:** 10.1530/EC-23-0253

**Published:** 2023-10-05

**Authors:** Tsung-Hui Wu, Guan-Yu Su, Tsung-Yun Liu, Hsiang-Tsui Wang, Chii-Min Hwu

**Affiliations:** 1 Section of Endocrinology and Metabolism, Department of Medicine, Taipei Veterans General Hospital, Taipei, Taiwan; 2 Institute of Food Safety and Health Risk Assessment, College of Pharmaceutical Sciences, National Yang Ming Chiao Tung University, Taipei, Taiwan; 3 Institute of Pharmacology, College of Medicine, National Yang Ming Chiao Tung University, Taipei, Taiwan; 4 PhD Program in Toxicology, Kaohsiung Medical University, Kaohsiung, Taiwan; 5 Faculty of Medicine, National Yang Ming Chiao Tung University School of Medicine, Taipei, Taiwan

**Keywords:** acrolein, acrolein protein conjugates, diabetic peripheral neuropathy, type 2 diabetes

## Abstract

Acrolein, an unsaturated aldehyde, plays a pathological role in neurodegenerative diseases. However, less is known about its effects on peripheral neuropathy. The aim of this study was to investigate the association of acrolein and diabetic peripheral neuropathy in patients with type 2 diabetes. We recruited 148 ambulatory patients with type 2 diabetes. Each participant underwent an assessment of the Michigan Neuropathy Screening Instrument Physical Examination. Diabetic peripheral neuropathy was defined as Michigan Neuropathy Screening Instrument Physical Examination score ≥ 2.5. Serum levels and urinary levels of acrolein protein conjugates were measured. Urinary acrolein protein conjugates-to-creatinine ratios were determined. Patients with diabetic peripheral neuropathy had significantly higher urinary acrolein protein conjugates-to-creatinine ratios than those without diabetic peripheral neuropathy (7.91, 95% CI: 5.96–10.50 vs 5.31, 95% CI: 4.21–6.68, *P* = 0.029). Logarithmic transformation of urinary acrolein protein conjugates-to-creatinine ratios was positively associated with diabetic peripheral neuropathy in univariate logistic analysis, and the association remained significant in multivariate analysis (OR = 2.45, 95% CI: 1.12–5.34, *P* = 0.025). In conclusion, urinary acrolein protein conjugates-to-creatinine ratio may act as a new biomarker for diabetic peripheral neuropathy in type 2 diabetes. The involvement of acrolein in the pathogenesis of diabetic peripheral neuropathy warrants further investigation.

## Introduction

Diabetic peripheral neuropathy (DPN) is the most common microvascular complication of type 2 diabetes mellitus (1). Most patients with DPN have large-fiber and small-fiber dysfunction (2). Peripheral neuropathy involving large fibers causes muscle weakness and sensory disturbances, and small fiber involvement is characterized by neuropathic pain, impaired temperature sensation, and autonomic dysfunction (2). While poor glycemic control and cardiovascular risk factors have been demonstrated to contribute to DPN, the pathogenetic mechanisms of DPN are multifactorial and complex (3). It has been proposed that exposure to environmental toxic agents may be associated with the development of DPN (4).

Acrolein is a highly reactive α,β-unsaturated aldehyde to which humans are exposed in many situations (5, 6, 7). Acrolein is generated from environmental sources such as engine exhaust, cigarette smoke, industrial waste, or combustion of organic substances (6). It can be released from overheated vegetable and animal fats (6). Furthermore, acrolein can be endogenously produced through myeloperoxidase, lipid peroxidation, and amine oxidase-mediated metabolism of polyamines (7). Acrolein exerts diverse toxic effects through various mechanisms, including DNA and protein adduction, disruption of mitochondria and endoplasmic reticulum, cell membrane damage, oxidative stress, and inflammation and immune dysfunction (8). Due to an abundance of polyunsaturated fatty acid without adequate antioxidant enzymes, neurons are vulnerable to oxidative stress and acrolein toxicity (6). Acrolein impairs mitochondrial function and increases endoplasmic reticulum stress in neuronal tissue (9). Acrolein can damage axonal membrane and myelin structure, resulting in neural injury (9). The overall neurotoxic effects of acrolein induce neuroinflammation and neurodegeneration and may play a role in the pathophysiology of peripheral neuropathy (10, 11).

Several studies have shown the association of acrolein with diabetes mellitus and its complications (11, 12, 13, 14, 15). Patients with diabetes mellitus had higher serum and urinary levels of acrolein adduct in comparison with healthy subjects (11, 12, 13). Evidence indicates that acrolein may contribute to diabetic retinopathy and diabetic nephropathy (14, 15). Recently, Yao *et al.* found that treatment with hydralazine, an acrolein scavenger, attenuated neuroinflammation and neuropathic pain in a rat model of diabetes (11). The authors proposed that acrolein might be involved in the pathogenesis of DPN (11).

Treatment of hyperglycemia alone has limited impacts on established DPN in type 2 diabetes (16). However, a number of disease-modifying agents for DPN have shown disappointing results (16). There is an unmet need for relevant biomarkers of DPN to facilitate drug development. Examination of the association between acrolein and DPN may lead to potential biomarkers and targeted treatments in the future. Therefore, the purpose of the present study was to investigate the association of acrolein and DPN in patients with type 2 diabetes. We hypothesize that exposure to acrolein is associated with the development of DPN in patients with type 2 diabetes.

## Methods

### Patients

Patients from the outpatient clinics at the Taipei Veterans General Hospital were eligible if they were at least 50 years of age and had type 2 diabetes mellitus for at least 1 year. Patients were excluded if they had an estimated glomerular filtration rate (eGFR, as calculated by the Modification of Diet in Renal Disease Study equation) below 15/min/1.73 m^2^ of body surface area or had a history of macrocytic anemia, severely affected acute stroke (National Institutes of Health Stroke Scale > 15), New York Heart Association Functional class III to IV congestive heart failure, muscular diseases, or peripheral arterial occlusive disease. Patients with a recent diagnosis of foot ulcers or nondiabetic neuropathy in the last 6 months were excluded as well. The ethics committee of the Taipei Veterans General Hospital approved the study protocol, and all participants provided written informed consent at the baseline examination.

### Measurements

After a 12-h overnight fast, participants received anthropometric assessment and blood pressure measurement at 08:00 h. An automated blood pressure recorder (HEM-7310, Omron Healthcare Inc., Kyoto, Japan) was used to determine the right arm blood pressure while each patient was seated. Height and weight were measured, and body mass index was calculated as weight in kilograms divided by the square of height in meters (kg/m^2^). Waist circumference was measured to the nearest millimeter with anthropometric tape around the umbilicus. Fasting blood samples were drawn for the measurements of glucose, glycated hemoglobin (HbA_1c_), lipids, biochemistry, and acrolein protein conjugates (Acr-PC). Urinary samples were obtained for the measurements of creatinine and Acr-PC. Urinary acrolein protein conjugates-to-creatinine ratios (Acr-PC/creatinine) were determined.

The Michigan Neuropathy Screening Instrument Physical Examination (MNSI-PE) was used for screening of DPN. The participants received physical assessment of lower extremity based on MNSI-PE, including evaluation of feet appearance, ulceration, ankle reflexes, vibratory perception using a 128 Hz tuning fork, and pressure perception using a 10 g monofilament. Abnormal findings were recorded and scored according to the MNSI guidelines. Diabetic peripheral neuropathy was defined as MNSI-PE score ≥ 2.5.

Data in relation to demographic features and physical activity were collected using an interview-administered questionnaire. The participants were asked to recall the amount of time per day for each of five levels of activity in a representative day within the past two weeks. The five levels of activity were basal (sleeping or lying down), sedentary (sitting or standing), slight (e.g., casual walking), moderate (e.g., aerobic dancing), and heavy (e.g., running). A physical inactivity score was calculated as sedentary hours divided by waking hours according to the formula reported previously (17).

Biochemical parameters including serum glucose, lipids, creatinine, and alanine aminotransferase (ALT) were measured using commercial assay kits (Roche Diagnostics) in an automatic clinical chemistry analyzer (Roche-Hitachi 7180, Roche Diagnostics). The measurement of HbA_1c_ was performed using capillary electrophoresis. Serum and urinary levels of Acr-PC were determined using the acrolein–lysine/cysteine/arginine adduct competitive enzyme immunoassay kit (Takara Bio. Inc.), and the details of detection methods were described elsewhere (18).

### Statistical analysis

Data are expressed as arithmetic mean ± s.d. for normally distributed continuous variables, geometric mean (95% CI) for non-normally distributed continuous variables, and number (percentage) for noncontinuous variables. Logarithmic transformation was used to reduce the skewness of fasting plasma glucose, HbA_1c_, ALT, triglyceride, serum and urinary Acr-PC levels, and urinary Acr-PC/creatinine levels prior to statistical analysis. Student’s *t*-test and *χ* ^*2*^ test were performed to compare the DPN group and non-DPN group for the continuous and categorical variables, respectively. Pearson correlation analyses were applied to investigate the association between DPN and serum Acr-PC levels, urinary Acr-PC levels, and urinary Acr-PC/creatinine levels. Univariate and multivariate logistic regression analyses were performed to analyze the association of different variables with DPN. A *P*-value less than 0.05 was considered statistically significant. All analyses were conducted using the Statistical Package for the Social Sciences Statistics Program (version 22.0, IBM Corporation, Armonk, NY, USA).

## Results

**Figure 1:**
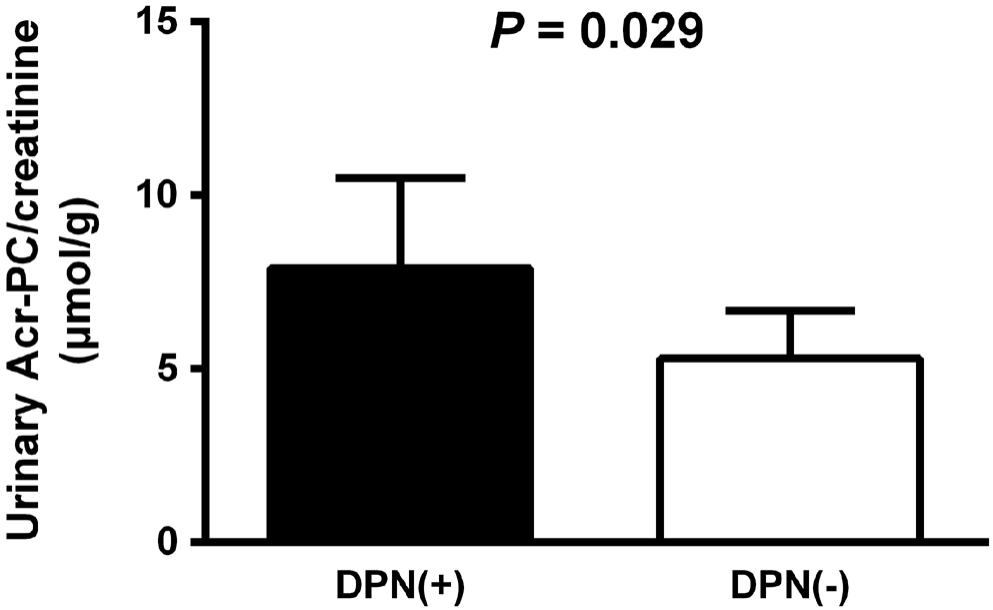
Geometric mean and 95% CI of urinary acrolein protein conjugates-to-creatinine ratio in patients with and without diabetic peripheral neuropathy. Student’s *t*-test on the logarithmic transformed data was used to compare the two groups. Urinary Acr-PC/creatinine, urinary acrolein protein conjugates-to-creatinine ratio; DPN, diabetic peripheral neuropathy.

A total of 148 patients were included in the current study. The clinical characteristics of the participants with and without DPN are listed in Table 1. Patients with DPN are older and had higher body mass index and waist circumference. The two groups of participants had comparable rates of smoking status, physical inactivity score, blood pressure, fasting plasma glucose, HbA_1c_, and lipid profiles. Patients with DPN had lower eGFR levels comparing to those without DPN. Serum levels and urinary levels of Acr-PC were not significantly different between the two groups. Urinary Acr-PC/creatinine levels were higher in the DPN group than in the non-DPN group (7.91 (5.96–10.50) in the DPN group, 5.31 (4.21–6.68) in the non-DPN group, *P* = 0.029; Fig. 1).

**Table 1:** Clinical characteristics of study participants with and without diabetic peripheral neuropathy.^a^

	DPN (+)	DPN (−)	*P*
*N*	65	83	
Age (years)	74.0 ± 9.3	69.0 ± 8.6	0.0010^b^
Men (*n*, %)	29 (45)	35 (42)	0.77
BW (kg)	66.1 ± 11.0	62.4 ± 11.1	0.046^b^
BMI (kg/m^2^)	26.2 ± 3.4	24.9 ± 3.5	0.029^b^
WC (cm)	92.8 ± 9.4	88.7 ± 9.2	0.010^b^
Physical inactivity score	0.61 ± 0.21	0.54 ± 0.23	0.051
Smoking (*n*, %)	10 (15)	22 (27)	0.10
SBP (mmHg)	143 ± 21	139 ± 20	0.27
DBP (mmHg)	78 ± 9	82 ± 11	0.071
Fasting plasma glucose (mg/dL)	129 (119–140)	121 (114–127)	0.18
HbA_1c_ (%)	7.7 (7.4–8.0)	7.3 (7.1–7.6)	0.069
eGFR (mL/min/1.73 m^2^)	63 ± 21	79 ± 27	<0.001^b^
ALT (U/L)	18 (16–21)	21 (19–23)	0.17
Total cholesterol (mg/dL)	151 ± 29	159 ± 27	0.086
LDL-C (mg/dL)	79 ± 22	83 ± 25	0.31
TG (mg/dL)	111 (98–124)	112 (101–124)	0.89
Serum Acr-PC (μmol/L)	68.68 (60.96–77.37)	74.84 (67.50–82.97)	0.28
Urinary Acr-PC (nmol/dL)	221.07 (173.85–281.12)	174.57 (146.16–208.48)	0.11

Data are expressed as arithmetic mean ± s.d. or geometric mean (95% CI) for continuous variables and number (percentage) for noncontinuous variables. Student's *t*-test and *χ^2^*test were used to compare the two groups.

^a^Diabetic peripheral neuropathy is defined as Michigan Neuropathy Screening Instrument Physical Examination score ≥ 2.5; ^b^*P* < 0.05.

Acr-PC, acrolein protein conjugates; ALT, alanine aminotransferase; BMI, body mass index; BW, body weight; DBP, diastolic blood pressure; DPN, diabetic peripheral neuropathy; eGFR, estimated glomerular filtration rate; HbA1c, glycated hemoglobin; LDL-C, low-density lipoprotein cholesterol; SBP, systolic blood pressure; TG, triglycerides; WC, waist circumference.

The results of the Pearson’s correlation analyses are summarized in Supplementary Table 1 (see section on supplementary materials given at the end of this article). There was no significant correlation between DPN and logarithmic transformation of serum Acr-PC levels and urinary Acr-PC levels. A significant but weak correlation was detected between logarithmic transformation of urinary Acr-PC/creatinine levels and DPN (r = 0.18, *P* = 0.029). The results of the logistic regression analyses are shown in Table 2. In univariate analyses, logarithmic transformation of urinary Acr-PC/creatinine levels, age, body mass index, waist circumference, and eGFR were associated with DPN. The association between logarithmic transformation of urinary Acr-PC/creatinine levels and DPN remained significant in multivariate analysis (OR = 2.45, 95% CI = 1.12–5.34, *P* = 0.025). Collinearity statistics were carried out and evaluated based on the variance inflation factors. All variance inflation factors (range 1.01–3.74) less than ten indicated no multicollinearity problems among the independent variables.

**Table 2:** Univariate and multivariate analysis of factors associated with diabetic peripheral neuropathy.^a^

	Univariate analysis		Multivariate analysis	
	OR (95% CI)	*P*	OR (95% CI)	*P*
Log(urinary Acr-PC/creatinine)	2.17 (1.06–4.42)	0.034^b^	2.45 (1.12–5.34)	0.025^b^
Age (years)	1.07 (1.02–1.11)	0.0020^b^	1.06 (1.01–1.10)	0.017^b^
Sex (men = 1, women = 0)	1.11 (0.57–2.13)	0.77		
BMI (kg/m^2^)	1.11 (1.01–1.23)	0.031^b^	1.04 (0.85–1.27)	0.70
WC (cm)	1.05 (1.01–1.09)	0.011^b^	1.04 (0.97–1.12)	0.32
Physical inactivity score	4.35 (0.98–19.29)	0.053		
Smoking	0.50 (0.22–1.16)	0.11		
SBP (mmHg)	1.01 (0.99–1.03)	0.27		
DBP (mmHg)	0.97 (0.94–1.00)	0.073		
Log(FPG)	6.25 (0.43–90.90)	0.18		
Log(HbA_1c_)	81.07 (0.68–9637.44)	0.071		
eGFR (mL/min/1.73 m^2^)	0.97 (0.96–0.99)	<0.001^b^	0.98 (0.96–1.00)	0.015^b^
Log(ALT)	0.32 (0.060–1.65)	0.17		
Total cholesterol (mg/dL)	0.99 (0.98–1.00)	0.087		
LDL-C (mg/dL)	0.99 (0.98–1.01)	0.31		
Log(TG)	0.89 (0.18–4.32)	0.89		

^a^Diabetic peripheral neuropathy is defined as Michigan Neuropathy Screening Instrument Physical Examination score ≥ 2.5; ^b^*P* < 0.05.

ALT, alanine aminotransferase; BMI, body mass index; DBP, diastolic blood pressure; eGFR, estimated glomerular filtration rate; FPG, fasting plasma glucose; HbA1c, glycated hemoglobin; LDL-C, low-density lipoprotein cholesterol; Log(ALT), logarithmic transformation of ALT (in U/L); Log(FPG), logarithmic transformation of FPG (in mg/dL); Log(HbA_1c_), logarithmic transformation of HbA_1c_ (in %); Log(TG), logarithmic transformation of TG (in mg/dL); Log(urinary Acr-PC/creatinine), logarithmic transformation of urinary Acr-PC/creatinine (in μmol/g); OR, odds ratio; SBP, systolic blood pressure; TG, triglycerides; urinary Acr-PC/creatinine, urinary acrolein protein conjugates-to-creatinine ratio; WC, waist circumference.

## Discussion

In this study, we found that urinary Acr-PC/creatinine levels were higher in patients with type 2 diabetes mellitus and DPN, compared with patients with type 2 diabetes mellitus without DPN. The association between urinary Acr-PC/creatinine levels and DPN remained significant after adjustments for clinical variables, including eGFR. These results suggest that urinary acrolein protein conjugates-to-creatinine ratio may be a potential surrogate biomarker of diabetic peripheral neuropathy in patients with type 2 diabetes mellitus.

Acrolein is one of the common environmental pollutants, and it is known to cause neurotoxicity and various neurological disorders (9). Oxidative stress is recognized as the main mechanism of acrolein-induced neurotoxicity (19). As a product and an initiator of lipid peroxidation, acrolein is considered as a perpetrator of oxidative stress (6). The *in vitro* and *in vivo* studies have identified that acrolein-induced oxidative damage can lead to mitochondrial and DNA dysfunction and exacerbate apoptosis (20). Acrolein also inhibits antioxidant enzymes and subsequently increases the rate of lipid peroxidation and oxidative stress (21). Acrolein is known to downregulate Nrf2, a key antioxidant regulator, and decreases capacity of antioxidant enzymes (22). Besides, acrolein exposure contributes to cellular deterioration by axonal membrane damage and myelin disruption (23). It represents another pathogenesis of functional deficits and cellular death triggered by acrolein (23).

Recent studies have shown an emerging role of acrolein-induced oxidative stress in the pathogenesis of diabetic complications (24, 25). Acrolein protein adducts are proposed to be associated with carbonyl stress in diabetic glomerular lesions (24). Acrolein compromises antioxidant defense system and mediates mitochondrial dysfunction in the diabetic retina (25). Acrolein is also involved in the neurodegenerative process in diabetic retinopathy (26). Besides, Yao *et al.* reported that treatment with acrolein scavenger hydralazine attenuated neuroinflammation and neuropathic pain in a rat model of diabetes (11). In our study, patients with type 2 diabetes mellitus and DPN had higher urinary Acr-PC/creatinine levels than non-DPN group. Urinary Acr-PC/creatinine levels is positively associated with occurrence of DPN after adjustments for clinical factors, including eGFR. Our results implicate that exposure to acrolein may increase the risk of peripheral neuropathy in patients with type 2 diabetes mellitus.

In the current study, we found a negative association between eGFR and DPN in patients with type 2 diabetes. In diabetic microvasculature, chronic intracellular hyperglycemia causes damage to vascular endothelium, leading to diabetic microvascular complications (27). Patients with DPN are more likely to have diabetic kidney disease (28). Meanwhile, patients with diabetic nephropathy tend to suffer from DPN and diabetic foot complications (28, 29). In our study, the correlation among the diabetic microvascular complications was compatible with previous studies. However, the association between DPN and urinary Acr-PC/creatinine levels kept significant after adjustments for clinical variables, including eGFR. Our results suggested urinary Acr-PC/creatinine level as an independent biomarker for DPN in patients with type 2 diabetes.

In this study, logistic regression analyses showed no significant association between DPN and parameters of glycemic control (Table 2). Improved glycemic control reduces incidence of DPN dramatically in patients with type 1 diabetes, but the beneficial effect becomes modest in patients with type 2 diabetes (1). The associations between DPN and blood glucose control in type 2 diabetes from different studies have been inconsistent (30, 31, 32). The Action to Control Cardiovascular Risk in Diabetes (ACCORD) trial showed decreased risk of DPN in intensive glycemic control group after 5 years of follow-up, but other large trials have shown no treatment effects (30, 31, 32). This discrepancy may be attributed to polypharmacy, concomitant comorbidities, hypoglycemia, and weight gain (1). A latent period of asymptomatic hyperglycemia before diagnosis of type 2 diabetes may also account for the inconsistent results (1). Further studies are needed to investigate the association between DPN and glycemic control.

In the present study, univariate analyses showed that occurrence of DPN is positively associated with age, body mass index, and waist circumference (Table 2). Older age is one of the well-documented risk factors for the development of diabetic peripheral neuropathy (33). The characteristics of patients with metabolic syndrome, including overweight and abdominal obesity, are associated with diabetic neuropathy in patients with type 2 diabetes as well (33). The pathophysiology of peripheral neuropathy associated with obesity includes altered lipid metabolism and inflammation, leading to microvascular and peripheral nerve injury (34). In our study, the results of the association between these well-known risk factors of peripheral neuropathy and DPN are compatible with previous studies. The association between DPN and urinary Acr-PC/creatinine levels kept significant after adjustments for the clinical risk factors.

There are some limitations in our study. First, the cross-sectional design of the study limits the ability to make casual relationships between variables, although it is beneficial in supporting the hypotheses. The lack of a control group prevents comparison with normal populations without diabetes as well. Second, the limited availability of analysis of acrolein renders its application in routine clinical practice. However, this noninvasive assessment of DPN can be easily applied in large epidemiological studies. This approach may also be used as an initial assessment of patients at high risk when the equipment is available. Last, serum and urinary levels of acrolein did not necessarily reflect the concentrations in target tissues. Further studies are required to explore acrolein-induced tissue injury in the peripheral nerve in experimental models of diabetes.

## Conclusions

In conclusion, urinary Acr-PC-to-creatinine ratio is associated with diabetic peripheral neuropathy in patients with type 2 diabetes mellitus after adjustments for clinical variables including estimated glomerular filtration rate. Urinary Acr-PC-to-creatinine ratio may be a biomarker for identification of patients with type 2 diabetes mellitus at high risk for diabetic peripheral neuropathy.

## Supplementary Materials

Table S1 Pearson correlation analysis between diabetic peripheral neuropathy‡ and serum levels of acrolein protein conjugates, urinary levels of acrolein protein conjugates, and urinary acrolein protein conjugates-to-creatinine ratio

## Declarations of interest

The authors declare no conflicts of interest in association with the present study.

## Funding

This work was supported by the National Science and Technology Council, Taiwan (MOST 110-2314-B-075-027-MY3), and the Taipei Veterans General Hospital (V109C-189, V110C-175, V111C-188, V112A-007). Dr. Hsiang-Tsui Wang was supported by the grants from National Health Research Institutes, Taiwan (NHRI-EX111-11027PI, NHRI-EX112-11027PI) and Ministry of Science and Technology, Taiwan (MOST-111-2320-B-A49-018). These funding agencies had no role in the study design, data collection, and analysis, decision to publish, or manuscript preparation. No organization provided funds to assist with the preparation of this article, and data analysis was not performed by employees of funders or any author who received funding. The funders did not offer writing support. The corresponding authors had complete access to all data in the study and the ﬁnal responsibility of the decision to publish.
