# Supplementary material for: Urinary acrolein protein conjugates-to-creatinine ratio is positively associated with diabetic peripheral neuropathy in patients with type 2 diabetes mellitus
Source: Endocr Connect. 2023 Oct 5;12(11):e230253. doi: 10.1530/EC-23-0253 (PMC10563594; doi:10.1530/EC-23-0253)
Supplement: Table S1 Pearson correlation analysis between diabetic peripheral neuropathy‡ and serum levels of acrolein protein conjugates, urinary levels of acrolein protein conjugates, and urinary acrolein protein conjugates-to-creatinine ratio [file supplementary_table_1.pdf]

**Table S1** Pearson correlation analysis between diabetic peripheral neuropathy‡ and serum levels of acrolein protein conjugates, urinary levels of acrolein protein conjugates, and urinary acrolein protein conjugates-to-creatinine ratio

| | r | *P* |
|---|---|---|
| Log(serum Acr-PC) | -0.090 | 0.28 |
| Log(urinary Acr-PC) | 0.13 | 0.11 |
| Log(urinary Acr-PC/creatinine) | 0.18 | 0.029* |

‡Diabetic peripheral neuropathy is defined as Michigan Neuropathy Screening Instrument Physical Examination score ≥2.5.

Acr-PC, acrolein protein conjugates; Log(serum Acr-PC), logarithmic transformation of serum levels of Acr-PC (in μmol/L); Log(urinary Acr-PC), logarithmic transformation of urinary levels of Acr-PC (in nmol/dL); urinary Acr-PC/creatinine, urinary acrolein protein conjugates-to-creatinine ratio; Log(urinary Acr-PC/creatinine), logarithmic transformation of urinary Acr-PC/creatinine (in μmol/g).

*$P < 0.05$
